# Utilizing physician modified fenestration on the castor branched stent technique for reconstruction of an isolated left vertebral artery on the aortic arch

**DOI:** 10.1038/s41598-024-54781-8

**Published:** 2024-02-19

**Authors:** Zeng-Rong Luo, Sai-Lan Li, Liang-Wan Chen, Rong-Da Huang

**Affiliations:** 1 Department of Cardiovascular Surgery, Fujian Medical University Union Hospital, Fuzhou, 350001 China; 2 Key Laboratory of Cardio-Thoracic Surgery (Fujian Medical University), Fuzhou, Fujian Province China; 3 Department of Cardiovascular Surgery and Cardiac Disease Center, Fujian Medical University Union Hospital, Fuzhou, 350001 China

**Keywords:** Vertebral artery, Thoracic endovascular aortic repair, Fenestration, Stents, Aneurysm, Aortic diseases

## Abstract

The study aimed to provide physician modified fenestration (PMF) on a single-branched stent for the aortic arch (Castor) to protect the isolated left vertebral artery (ILVA) during thoracic endovascular aortic repair (TEVAR). Patients who underwent TEVAR involving ILVA reconstruction through PMF performing on the Castor branched stent were included in a retrospective, multi-centre study from June 2018 to December 2022. In these patients, all proximal landing zones of "Castor" were positioned in Ishimaru zone 2a. A total of twenty-five patients met the inclusion criteria and the achievement rate showed 25/25 (100%) success in them. The twenty-five patients had a median follow-up length of 28.5 ± 14.6 months. One patient (4.0%) suffered from postoperative ischemic stroke before discharge. One patient (4.0%) died from a hemodialysis-related brain hemorrhage before discharge on the 29th day after the procedure. One patient died of advanced liver cancer in the 33th month after discharge. Aortic rupture, stroke or spinal cord injury did not occur throughout the follow-up period after discharge. Two patients (8.0%) experienced endoleak at the fenestration, however, resulting in only one’s necessity for reintervention. Notably, the procedure effectively maintained ILVAs patency for all patients during follow up. According to our preliminary findings, performing a TEVAR under local anaesthesia using PMF on a Castor branched stent for ILVA preservation appeared practical, secure, and effective.

## Introduction

The vertebral artery is a vital blood channel nourishing the brain^1^. The second most frequent anomaly in the setting of aortic arch variants is the isolated left vertebral artery (ILVA), which arises straight from the aortic arch and is situated between the left common carotid artery (LCCA) and the left subclavian artery (LSA). ILVA is a common accompaniment to about 3.8% of individuals with aortic dissection^2–4^.

In most cases, the origin variation of the vertebral artery does not result in overt clinical complaints. But to provide a suitable proximal sealing length when confronted with this anomaly during thoracic endovascular aortic repair (TEVAR), surgeons usually have to cover the ILVA^5^. Improper dispose of ILVA might result in posterior cerebral ischemia, infarction, or spinal cord ischemia, especially when facing an incomplete circle of Willis^6,7^.

According to current recommendations, the ILVA repair techniques are yet uncertain. The application of ILVA transposition has shown positive outcomes^8^. Improved safety and less invasiveness are benefits of total endovascular repair of ILVA using physician modified fenestration (PMF) or in situ fenestration (ISF) on the conventional stent^9^. However, we believe that there is still room for improvement in these two approaches. First, the PMF technique employed a conventional stent with a "bare area" in the proximal landing zone, which might increase the risk of retrograde type A aortic dissection. Second, the main challenge of ISF lies in puncture rupture of stent membrane, which depends on the angle of ILVA and the aortic arch. When encountering a large angle between ILVA and aortic arch, the puncture system is difficult to fix to the aortic arch stent, and there is a potential risk of failure to puncture rupture of stent membrane and aortic injury.

Castor single-branched stent graft (MicroPort Medical, Shanghai, China) was designed with a branch section to retain the LSA while sealing entry tearings. In this context, we introduced our preliminary experience and short-term outcomes of TEVAR conducted under local anaesthesia, employing the PMF on Castor branched stent technique with proximal landing zone located in Ishimaru zone 2a for patients accompanying with an ILVA.

## Methods

### Clinical cases and methodology

This multi-center retrospective study comprised twenty-five patients from three medical centres who underwent thoracic endovascular aortic repair (TEVAR) with ILVA reconstruction via *in-vitro* fenestration (PMF) on Castor branched stent technique with proximal landing zone of "Castor" located in Ishimaru zone 2a. The study period spanned from June 2018 to December 2022.

The criteria for inclusion in this study encompassed the following aspects: (1) Patients with ILVA underwent total endovascular repair and LVA dominance; (2) The distance from lesion to LCCA is greater than 1.5 cm; the distance from lesion to ILVA is less than 1.5 cm; in other words, ILVA required reconstruction while the left common carotid artery (LCCA) did not necessitate reconstruction (namely, utilizing the "Castor" landing in zone 2a), and (3) The diameter of ILVA is larger than 2 mm; ILVA was reconstructed via PMF on Castor branched stent technique. In our centre, the indication of TEVAR intervention was assessed by a multidisciplinary panel. ILVA reconstruction was performed in patients with a dominant ILVA or symmetric vertebral arteries assessed by two experienced radiologists. We did not perform reconstruction of ILVA via *in-situ* fenestration (ISF) to avoid neck incisions and perform local anaesthesia procedures. ILVA reconstruction was not performed in patients with predominant right vertebral artery unless they concurrently received TEVAR and EVAR to prevent spinal cord ischemia (SCI). Patients were excluded if: (1) without ILVA; (2) underwent TEVAR without ILVA reconstruction, or (3) ILVAs were reconstructed via open surgery or hybrid procedure.

Preoperative computed tomography angiography (CTA) with 3-dimensional reconstructions were performed on all patients using a Revolution CT with a scanning thickness of 0.625 mm. Imaging-qualified DICOM data was passed to the centre's two experienced senior radiologists for joint interpretation and analysis. For this procedure, a landing zone of at least 1.5 cm away from proximal end of aortic lesions along the outer curvature of aortic arch had been designated.

### Ethic statements

The Declaration of Helsinki was followed when conducting the research. The ethics committees of Fujian Medical University Union Hospital, Fujian Medical University Longyan First Hospital and Fujian Medical University Nanping First Hospital approved the study (2023KY167, date: 2023-03-30) and waived the requirement for informed consent due to the retrospective nature of this study.

### In-vitro fenestration for isolated left vertebral artery

**Figure 1:**
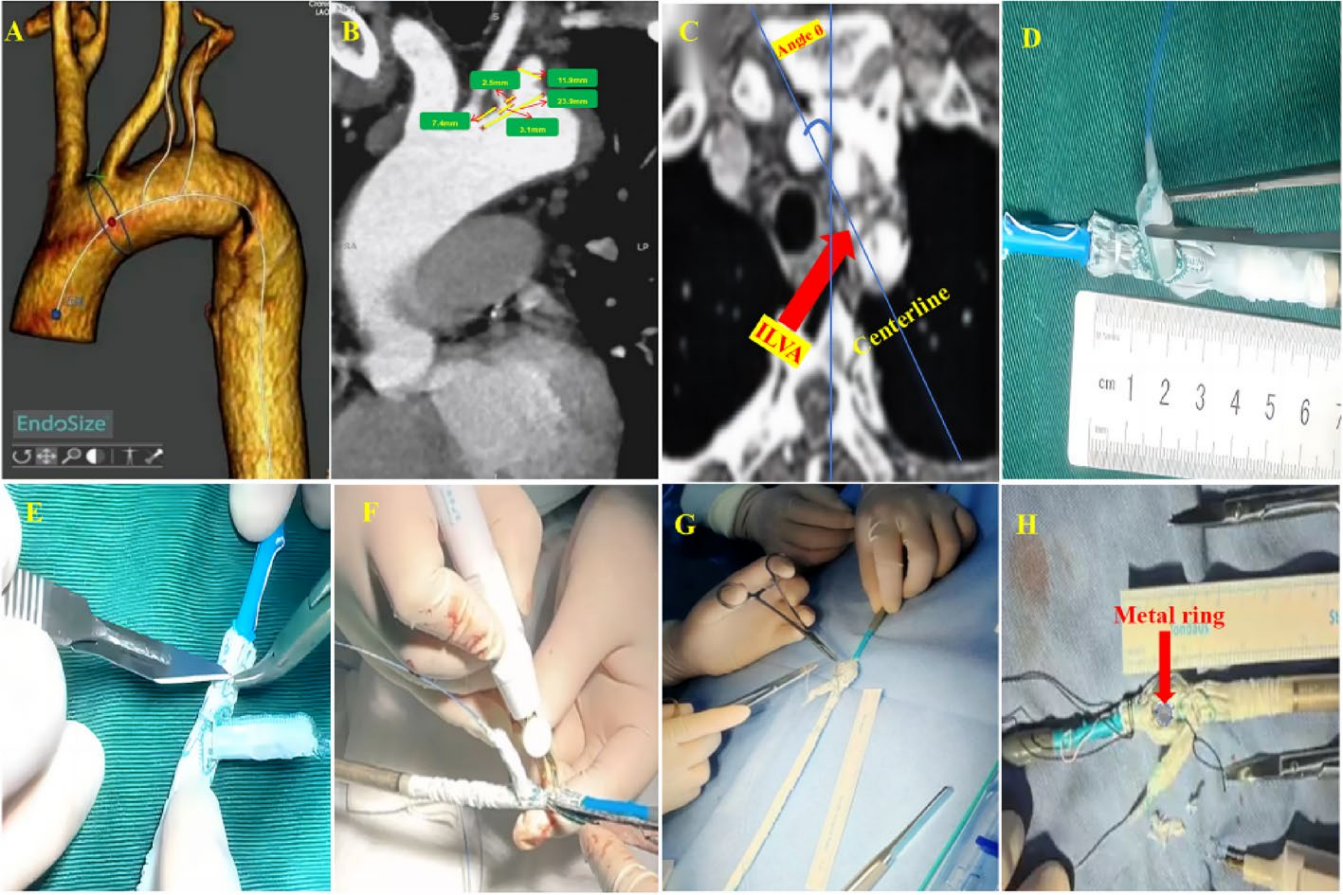
(**A**–**C**) Accurate measurements of the import parameters based on the CTA using EndoSize software before the PMF of ILVA. (**D**–**H**) The PMF of ILVA was performed on the Castor main stent. *CTA* computed tomography angiography, *PMF* physician modified fenestration, *ILVA* isolated left vertebral artery.

Single physician modified fenestration (PMF) was conducted to reconstruct the isolated left vertebral artery (ILVA) in our study. As the design of the fenestration was crucial, precise measurements should be conducted to determine the location of the ILVA fenestration before the procedure. The measured parameters included aortic diameter, arch angle, branch diameter, branch spacing and angle. These measurement data could be obtained by analyzing pre-operative CTA using EndoSize software (Fig. 1A–C). They allowed the protocol for PMF to be designed. Meanwhile, based on the PMF design plan, the exact spacing of the PMF and the branch stent was established. Next, we proceeded with the PMF process. We first used a blade to make a small opening at the positioning point, then used an electric knife to trim the size and shape of the fenestration window. To minimize the possible damage to to the textile graft material, we finally reinforced the fenestration window using a metal ring. Then the modified Castor stent was reinstalled into the delivering system when the PMF design was finished. An effort was made to prevent deformation and truncation. Additionally, the left subclavian artery (LSA) was reconstructed by the Castor branch stent. The process of performing ILVA physician modified fenestration on the Castor main graft was shown in (Fig. 1D–H). When no intraoperative endoleak and migration of stent or fenestration were observed, to reduce excessive operations, we did not use a bridging stent graft for the ILVA after reinforcing the fenestration window using a metal ring.

Following the reconstructive process, we developed guidelines for clopidogrel (75 mg qd) mono-antiplatelet treatment for at least six months.

### Post-observation

Documentation on demographics, anatomy, intra-operative and post-operative parameters were logged. Before procedure and discharge, computed tomography angiography (CTA) was performed on each individual. CTA were followed up at 1, 3, and 12 months and then once a year thereafter. The subsequent clinical data was gathered through telephone interviews and hospital visits.

### Definition and outcomes

Technical success was determined by the Castor stent being deployed precisely, by the IVF matching more than 80% of the ILVA, by the absence of conversion to open repair or death within 24 h, and by the absence of type I or III endoleak in the course of angioplasty. Major adverse events (MAEs) were composed of aortic-related mortality, aortic rupture, stroke, spinal cord injury, puncture site infection, myocardial infarction, severe pneumonia, acute kidney injury and other severe sequelae. Patency was determined to be a target of fifty percent or less vascular stenosis^9^.

Guidelines from Renal Disease Improving Global Outcomes (KDIGO) were used to determine acute kidney injury^10^. Endoleaks were diagnosed as constant contrast material flow from the graft or aneurysm sac^11^.

### Statistical analysis

The collected data were statistically analyzed using SPSS Version 26.0 (SPSS Inc., Chicago, USA). Shapiro–Wilk test results were employed to determine the distribution of data. The median and interquartile values were utilized for non-normally distributed continuous statistics, whereas the mean, standard deviation (SD) was used for normally distributed continuous variables. The terms number and percentage were used to denote categorical variables.

## Results

We have investigated twenty-five patients during the period between June 2018 and December 2022 in this retrospective study. All twenty-five patients were diagnosed with thoracic aortic disease (TAD) and underwent TEVAR and ILVA reconstruction utilizing physician modified fenestration (PMF) on the Castor branched stent technique with the proximal landing zone of "Castor" located in Ishimaru zone 2a. The study population displayed a predominant male composition (84.0%) with an average age of 62.5 ± 9.8 years and an average body mass index (BMI) of 24.9 ± 4.5 kg/m^2^. Among these patients, eighteen (72.0%) had hypertension, seven (28.0%) had diabetes, and three (12.0%) had hyperlipidemia. Eighteen (72.0%) had a history of tobacco abuse, six (24.0%) had a history of drinking, three (12.0%) suffered from acute kidney injury, and one (4.0%) had a history of liver cancer. None suffered from paraplegia, ischemic stroke or peripheral vascular disease. Regarding the lesion types, there were seventeen (68.0%) Stanford-type B dissection, three (12.0%) penetrating aortic ulcer, and five (20.0%) thoracic aortic aneurysms.

Eighteen (72.0%) and seven (28.0%) patients accompanied with left VA dominance and symmetric VA, whereas none (0.0%) patient accompanied with right VA dominance. The ILVAs entered the Willis circle to form the basilar artery and emitted the posterior-inferior cerebellar artery in all patients. The ILVAs' diameters were 3.7 ± 0.6 mm, and the proximal extensions of the treated lesion were 23.1 ± 1.6 mm. Table 1 contains demographic details and baseline clinical information.

**Table 1:** Baseline characteristics of 25 patients.

Variable	N (%) or mean ± SD
Demographics	
Age (years)	62.5 ± 9.8
Male	21 (84.0%)
BMI (kg/m^2^)	24.9 ± 4.5
Tobacco abuse, n (%)	18 (72.0%)
Drinking, n (%)	6 (24.0%)
Comorbidities, n (%)	
Hypertension	18 (72.0%)
Diabetes	7 (28.0%)
Hyperlipidemia	3 (12.0%)
AKI	3 (12.0%)
Peripheral vascular disease	0 (0.0%)
History of tumor	1* (4.0%)
Type of pathologies (n, %)	
Type B aortic dissection	17 (68.0%)
Acute dissection	14 (56.0%)
Chronic dissection	3 (12.0%)
Aortic arch aneurysm	5 (20.0%)
Penetrating aortic ulcer	3 (12.0%)
Type of presenting disease (n, %)	
Chest-back pain	21 (84.0%)
Hoarseness + Cough	1 (4.0%)
Dizziness	1 (4.0%)
Asymptomatic	2 (8.0%)
Vertebral artery dominance	
LVA dominance	18 (72.0%)
RVA dominance	0 (0.0%)
Symmetric VA	7 (28.0%)
Imaging parameters	
Proximal landing zone diameter, mm	31.0 ± 3.1
ILVA diameter, mm	3.7 ± 0.6
LSA diameter, mm	10.3 ± 1.4
LCCA-LSA distance, mm	15.5 ± 3.6
ILVA-LSA distance, mm	4.6 ± 1.8
The treated lesion-LSA distance	2.8 ± 1.8
The proximal extension of the treated lesion, mm	23.1 ± 1.6
Complete circle of Willis	3 (12.0%)
ILVA calcification	8 (32.0%)
No ILVA calcification	17 (68.0%)
ILVA entering the circle of Willis to form the basilar artery	25 (100%)
ILVA sending off the posterior-inferior cerebellar artery	25 (100%)

*BMI* body mass index, *AKI* acute kidney injury, *LVA* left vertebral artery, *RVA* right vertebral artery, *VA* vertebral artery, *ILVA* isolated left vertebral artery, *LSA* left subclavian artery, *LCCA* left common carotid artery.

*Liver cancer.

### Procedural data

All patients underwent TEVAR with ILVA and LSA reconstruction via PMF on Castor branched stent technique with the proximal landing zone of "Castor" precisely situated in Ishimaru zone 2a. A 100% technical success rate was attained. Fifty target vessels (TVs) were successfully reconstructed, including twenty-five ILVAs utilizing PMF and eighteen bridging stents were deployed in them, twenty-five LSAs via Castor branched stents. The mean procedure time was 74.5 (66.5, 79.5) mins and the median volume of contrast material was 114.0 (106, 120) ml. The median hospital stay was 5.0 (4.3, 6.8) days. The mean proximal Castor mainbody’s diameter was 34.0 ± 3.9 mm and oversize rate was 9.5 ± 2.7%; the median Castor branche’s diameter was 11 (10,12) mm and oversize was 0.5 ± 0.5 mm. No migration of stent or fenestration was observed. One intraoperative endoleak occurred and the endoleak disappeared after implantation of bridging stent (Table 2).

**Table 2:** The surgical data of the 25 patients.

Variable	N (%) or mean ± SD, median (Q1, Q3)
Type of reconstruction (n, %)	
Castor + PMF	25 (100.0%)
Local anaesthesia (n, %)	25 (100.0%)
Success (n, %)	
LSA success	25 (100.0%)
ILVA success	25 (100.0%)
Bridging stent information	
With bridging stent	18 (72.0%)
Fluency (BD, USA)	11 (44.0%)
diameter, mm	4.1 ± 1.2
Length, mm	25.4 ± 6.0
Apollo (Microport, Shanghai, China)	7 (28.0%)
Diameter, mm	4.2 ± 1.1
length, mm	26.8 ± 5.5
Without bridging stent	7 (28.0%)
Operation time (mins)	74.5 (66.5, 79.5)
Volume of contrast material, ml	114.0 (106, 120)
Stents brand and size	
Proximal Castor mainbodys diameter, mm	34.0 ± 3.9
Proximal Castor mainbodys diameter oversize rate (%)	9.5 ± 2.7
Castor branches diameter, mm	11 (10,12)
Castor branches diameter oversize, mm	0.5 ± 0.5
Castor branches length, mm	37.5 (35,40)
Fenestration- LSA distance	4.6 ± 1.8
Simultaneous abdominal endovascular treatment (n, %)	3 (12.0%)
Endoleak (n, %)	0* (0.0%)
Hospital stay (days)	5.0 (4.3, 6.8)

*PMF* physician modified fenestration, *ILVA* isolated left vertebral artery, *LSA* left subclavian artery.

One case of endoleak disappearance after intraoperative implantation of bridging stent.

### In-hospital outcome

**Figure 2:**
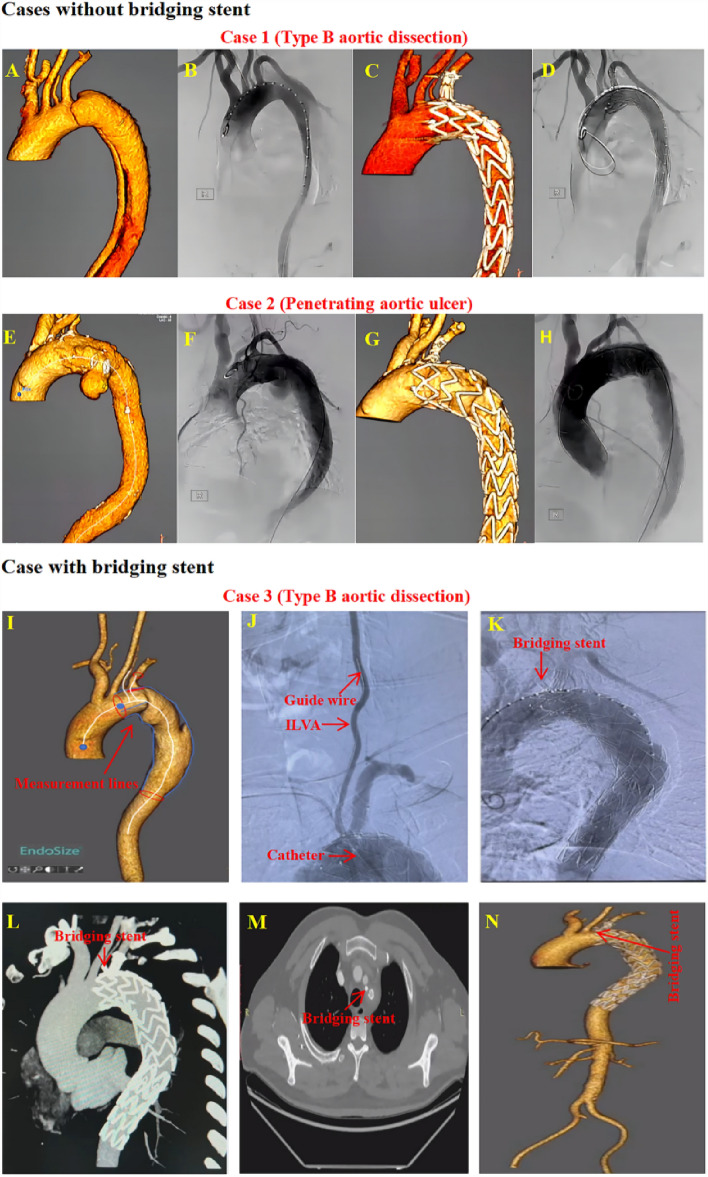
Presentations of outcomes using PMF on Castor branched stent technique to reconstruct ILVAs. Two cases without bridging stent implantation: (**A**,**E**) CTA display the aortic pathologies. (**B**,**F**) DSA show the aortic pathologies. (**C**,**G**) CTA display the perfect procedure utcomes. (**D**,**H**) DSA show the perfect procedure outcomes. One case with bridging stent implantation: (**I**) Measurement lines of preoperative CTA using EndoSize software (version 3.1.36 (5bb19e4)). (**J**) Intraoperative implementation of rebuilding ILVA. (**K**) DSA display the perfect procedure outcomes. (**L**–**N**) CTA display the perfect procedure outcomes. *PMF* physician modified fenestration, *ILVA* isolated left vertebral artery, *CTA* computed tomography angiography, *DSA* digital subtraction angiography.

No death occurred during the procedure. One patient (4.0%) suffered from postoperative ischemic stroke before discharge. One patient (4.0%) died from a hemodialysis-related brain hemorrhage before discharge on the 29th day after the procedure, who required hemodialysis due to acute kidney injury existing before the procedure. Meanwhile, this patient also suffered from in-hospital pulmonary infection. In addition, one patient (4.0%) developed ischemic symptoms of the left arm because the small brachial artery appeared relatively narrow after tying knots in this patient with short stature. No MAEs including aortic rupture, spinal cord injury, puncture site infection, myocardial infarction, and other severe adverse events were observed. No patient received reintervention before discharge. Except for the absence of CTA follow-up of the in-hospital death due to brain hemorrhage, no endoleak was found by CTA review before discharge. All TVs were patent without occlusion/ stenosis or migration of Castor stent or physician modified fenestration (see Table 3, Fig. 2 and Supplemental videos [A,A’]).

**Table 3:** In-hospital complications of the 25 patients.

Variable	N (%)
Aortic rupture (n, %)	0/25 (0.0%)
SCI (n, %)	0/25 (0.0%)
Ischemic stroke (n, %)	1/25 (4.0%)
Cerebral hemorrhage (n, %)	1*/25 (4.0%)
Access vessel complication (n, %)	0/25 (0.0%)
Puncture site infection (n, %)	0/25 (0.0%)
Ischemic symptoms of the left arm (n, %)	1/25 (4.0%)
Myocardial infarction (n, %)	0/25 (0.0%)
Severe pneumonia (n, %)	1*/25 (4.0%)
AKI (n, %)	3*/25 (12.0%)
ILVA instability (n, %)	0/25 (0.0%)
In-hospital aortic-related mortality (n, %)	0/25 (0.0%)
In-hospital mortality (n, %)	1*/25 (4.0%)

*SCI* spinal cord injury, *AKI* acute kidney injury, *ILVA* isolated left vertebral artery.

*These events occurred on a same patient.

### Follow up

**Figure 3:**
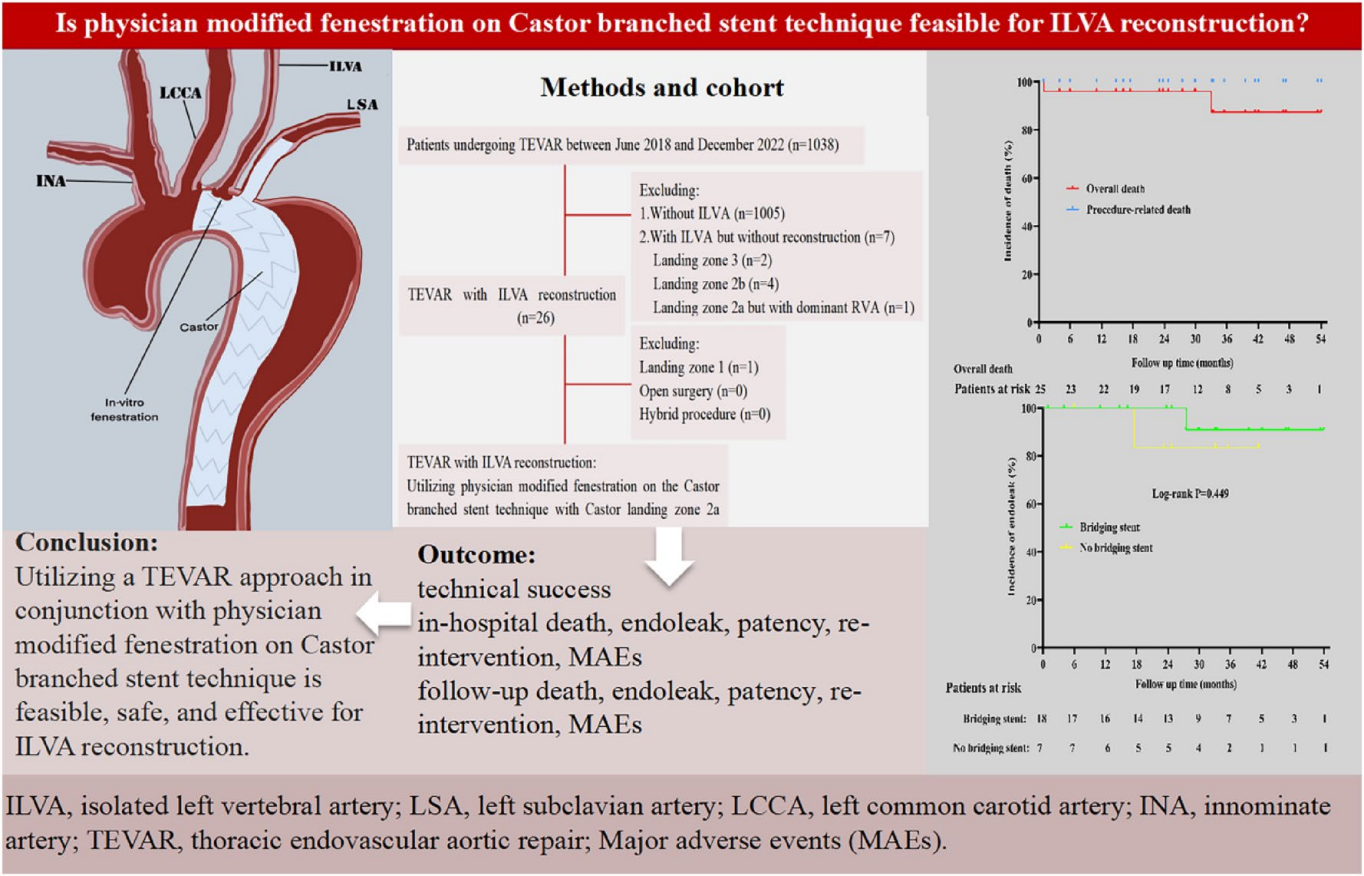
An illustrative summary of our findings.

The twenty-five patients had an average follow-up duration of 28.5 ± 14.6 months. Apart from one hospital death, twenty-four surviving patients discharged from the hospital. During follow up period, one patient died of advanced liver cancer in the 33th month after discharge. As mentioned earlier, the ischemic symptoms of the left arm and ischemic stroke disappeared. In addition to the previously mentioned in-hospital death combined with AKI, the renal function of the other two AKI patients recovered completely without undergoing CRRT. Except for two revascularized TVs of the in-hospital death without CTA follow-up, all the forty-eight successfully revascularized TVs remained patent. Two patients experienced endoleak at the fenestration after discharge, including 1 case with bridging stent and 1 case without bridging stent. There was no significant difference in the incidence of endoleak between the cases with bridging stent and the cases without bridging stent (Log-rank P = 0.449) (Fig. 3). The endoleak of the case without bridging stent underwent re-intervention by implanting a bridging stent, while the endoleak of the case with bridging stent was dealed with follow-up instead of re-intervention. The endoleak of these two cases completely disappeared during the next examination. Throughout the follow-up, no severe adverse event including aortic rupture, spinal cord injury, P-SINE, D-SINE, migration of stents or IVF and occlusion of LSA or ILVA was observed during follow up (Table 4 and Fig. 3).

**Table 4:** Follow-up information of the 24 survival patients after discharge.

Variable	N (%)
Aortic rupture (n, %)	0/24 (0.0%)
SCI (n, %)	0/24 (0.0%)
Stroke (n, %)	0/24 (4.0%)
Ischemic symptoms of the left arm (n, %)	0/24 (0.0%)
P-SINE (n, %)	0/24 (0.0%)
D-SINE (n, %)	0/24 (0.0%)
Endoleak (n, %)	2/24 (8.3%)
Migration of stents or fenestration (n, %)	0/24 (0.0%)
Occlusion of LSA or ILVA (n, %)	0/24 (0.0%)
Secondary intervention (n, %)	1/24 (4.2%)
Follow-up aortic-related mortality (n, %)	0/24 (0.0%)
Follow-up overall mortality (n, %)	1*/24 (4.2%)

*SCI* spinal cord injury, *P-SINE* proximal stent graft-induced new entry, *D-SINE* distal stent graft-induced new entry, *ILVA* isolated left vertebral artery, *LSA* left subclavian artery.

*Died of advanced liver cancer.

## Discussion

ILVA greatly influences strategies for reconstructing the aortic arch^12^. It is crucial to manage the condition carefully to avoid posterior circulation ischemia, ischemic stroke, and spinal cord injury (SCI). Reconstruction of the ILVA has been suggested as an alternative to direct coverage based on considerations such as dominance, vertebral artery symmetry, and circle of Willis completeness^13^. Yang et al. proposed rebuilding the ILVA because just 27% of Chinese people had a complete Willis circle^8,14^. Furthermore, Piffaretti et al. claimed that in individuals who experienced prior extensive aortic covering, retention of ILVA can lower the chance of SCI^15^.

The aortic arch lesions and ILVAs can be treated in many ways, including complete surgical treatment^12^, hybrid operations^8,15^, and parallel stent approaches^16^. Yet, since there was little pertinent research, there was no agreement on the approach for ILVA reconstruction up to this point. Currently, the total open surgery possesses a significant trauma^12^, hybrid procedure needs dissection and anastomosis of the target vessels, and many potential complications such as nerve injury, vocal cord paralysis, lymphatic leakage are also worthy of attention^8,15^. The problem of endoleak and the long-term patency are worrying after the parallel stents technique^17^.

Recently, the reported *in-situ* fenestration (ISF) and physician modified fenestration (PMF) techniques on conventional unbranched stents were suggested as an approach to manage ILVA, suggesting possible substitutes with promising short-term outcomes^9^. Nevertheless, the potential for a stent or PMF migration remains a notable concern^18^. Yet, as with the reconstruction of the left carotid common artery (LCCA), the reconstruction of ILVA by ISF requires an incision in the neck to dissect ILVA and is affected by the curvature or angle variation of ILVA^19^. Additionally, obtaining effective fenestration with the ISF approach demands a temporary ILVA blood flow cut-off, which may increase the risk of posterior circulation ischemia or SCI.

In our study, we introduce the application of PMF on the Castor branched stent technique to reconstruct ILVAs to optimize these deficiencies simultaneously. Based on our prior experience utilizing this approach to repair branches of the aortic arch^20^, PMF on Castor branched stent technique was recommended for our research patients who needed reconstruction of both the ILVA and left subclavian artery (LSA).

The advantages of our investigation can be summarized in three points. Our procedures were primarily conducted under local anaesthesia as the cervical incisions can be circumvented. This approach mitigated complications associated with mechanical ventilation and yielded cost savings for the hospital. Secondly, even in instances where blood flow through the PMF for ILVA was not patent enough, we could deploy a bridging stent or coil through the PMF orifice via a transfemoral route. This obviated the need for cervical incisions or punctures and prevented damage to the ILVA. Furthermore, this technique remained impervious to variations in ILVA curvature or angle, ensuring ample ILVA blood supply via the PMF, thereby diminishing the risk of posterior circulation ischemia or spinal cord injury (SCI).

Nonetheless, particular concerns PMF technique. Notably, modifications to the stent could compromise its stability and long-term durability^21^. Addressing these concerns, PMF on the Castor branched stent technique offers solutions. First, to minimize the possible damage to to the textile graft material, we reinforced the fenestration window using a metal ring. Second, the inherent stability of the Castor material mitigates instability risks posed by PMF. Third, Castor's branched architecture provides enhanced anchorage, minimizing the possibilities of stent or PMF migration^22^. Notably, Castor's branches can function as reference points, facilitating optimal PMF alignment with the ILVA ostium.

Our early experience showed that on the basis of the above conveniences, this approach could still achieve high technical success without procedure-related death, aortic rupture or other significant adverse problems. Except for a cerebral haemorrhage unrelated to the procedure that caused one patient to die, this procedure also produced positive perioperative. Moreover, even in cases without a bridging stent, the patency rate maintained 100% during follow up, demonstrating an acceptable long-term patency rate. In addition, continuous blood flow through ILVA during the procedure might have contributed to the lack of cerebral infarction or SCI observed in all follow-up individuals.

Still, some researchers believe that the PMF aperture needs to be filled by bridging stent, which can prevent the occurrence of endoleak or branch artery occlusion^23^. While further research suggests that even bridging stents show signs of weakness and lack of stability in ISF procedures^24^. To step back, even the implantation of the bridging stent was needed during our procedure, we could effortlessly implant it through the anterograde PMF aperture via the transfemoral path. Compared with the retrograde path of ISF and chimney, this technique appeared less invasive. In this study, 8 out of 25 cases were not implanted with bridging stents due to calcification in ILVA. One patient with bridging stent and one patient without bridging stent experienced endoleak at the fenestration after discharge, with no significant difference in the incidence of endoleak (Log-rank P = 0.449). The endoleak of the case without bridging stent underwent re-intervention by implanting a bridging stent effortlessly through the PMF aperture in an anterograde way, while the endoleak of the case with bridging stent was dealed with follow-up instead of re-intervention. The endoleak of these two cases completely disappeared during the next examination. Therefore, we advocate that PMF on Castor stent is a practical and less intrusive option with favorable postoperative results.

### Limitations

The study has certain limitations. First, although this study was a multi-center study, it was a small population study due to the low incidence of ILVA. Second, the possible impact of the surgeon's experience on the surgery results must also be considered. Considering this reason, it is advised to interpret the patency rate and endoleak rate cautiously. Further research with larger cohorts will be pivotal in validating the efficacy of this technique and expanding our understanding of its long-term implications.

## Conclusions

In conclusion, our study presents valuable insights into the feasibility, safety, and effectiveness of utilizing a TEVAR approach under local anaesthesia in conjunction with physician modified fenestration (PMF) on Castor branched stent technique for isolated left vertebral artery (ILVA) reconstruction. Despite the limitations acknowledged, our preliminary findings highlight the practicality and success of this approach. This initial experience underscores the potential of our approach in maintaining ILVA patency and minimizing adverse events, contributing to the evolving landscape of aortic arch reconstruction methods.

### Supplementary Information

Supplementary Video 1.

Supplementary Video 2.

## Data Availability

The data that support the findings of this study are available from Fujian Cardiac Medical Center but restrictions apply to the availability of these data, which were used under license for the current study, and so are not publicly available. Data are however available from Rong-Da Huang author upon reasonable request and with permission of Fujian Cardiac Medical Center.

## Abbreviations

BMI: Body mass index
PMF: Physician modified fenestration
SCI: Spinal cord injury
AKI: Acute kidney injury
ILVA: Isolated left vertebral artery
LSA: Left subclavian artery
LCCA: Left common carotid artery
TEVAR: Thoracic endovascular aortic repair
CTA: Computed tomographic angiography
